# Evaluation of the RHAM-based Pluslife Rapid SARS-CoV-2 assay and comparison with EasyNAT Rapid SARS-CoV-2 assay and Wondfo 2019-nCoV antigen assay

**DOI:** 10.1128/spectrum.03418-24

**Published:** 2025-10-20

**Authors:** Guosheng Zhong, Zhiwei Zhao, Yuting He, Xuegao Yu, Tingting Yang, Huanliang Liu, Shibing Li, Cha Chen, Mingyong Luo, Bin Huang

**Affiliations:** 1 Department of Laboratory Medicine, The First Affiliated Hospital, Sun Yat-sen University 26469 , Guangzhou, China; 2 Department of Clinical Laboratory, The Sixth Affiliated Hospital, Sun Yat-sen University 26469 , Guangzhou, China; 3 Department of Clinical Laboratory, Guangdong Women and Children Hospital 90405 , Guangzhou, China; 4 Women and Children’s Hospital, Southern University of Science and Technology 90405 , Guangzhou, China; 5 Department of Laboratory Medicine, The Second Affiliated Hospital, Guangzhou University of Chinese Medicine 47879, Guangzhou, China; Institut National de Santé Publique du Québec, Sainte-Anne-de-Bellevue, Quebec, Canada

**Keywords:** SARS-CoV-2, isothermal amplification, POCT, RT-qPCR, Pluslife Rapid SARS-CoV-2 assay, EasyNAT Rapid SARS-CoV-2 assay, Wondfo 2019-nCoV antigen assay

## Abstract

Severe acute respiratory syndrome coronavirus 2 (SARS-CoV-2) remains a significant public health challenge. This study evaluated the Pluslife SARS-CoV-2 assay employing proprietary RNase Hybridization-Assisted amplification (RHAM) isothermal amplification technology, with comparative performance assessment against the EasyNAT Rapid SARS-CoV-2 assay and the Wondfo 2019-nCoV antigen assay. Analytical testing determined the Pluslife assay’s limit of detection to be 400 copies/mL, with no cross-reactivity against common respiratory viruses and no interference from tested substances. Using RT-qPCR as the reference standard on 197 nasopharyngeal swabs, the Pluslife assay showed a positive percentage agreement (PPA) of 96.30% and a negative percentage agreement (NPA) of 99.30% (*κ* = 0.962, *P* < 0.001). The EasyNAT assay demonstrated a PPA of 96.30% and NPA of 98.60% (*κ* = 0.949, *P* < 0.001). The Wondfo antigen assay showed a PPA of 81.48% and NPA of 100% (*κ* = 0.865, *P* < 0.001). The Pluslife assay demonstrates comparable diagnostic accuracy to EasyNAT for SARS-CoV-2 detection, while offering advantages in portability, speed, and cost. Its performance significantly exceeded that of the Wondfo antigen assay, particularly in reducing false negatives. The Pluslife Mini Dock platform shows potential for rapid SARS-CoV-2 screening in diverse settings.

**IMPORTANCE:**

The newly developed Pluslife Rapid severe acute respiratory syndrome coronavirus 2 (SARS-CoV-2) assay is a next-generation molecular point-of-care testing (POCT) method, based on RNase Hybridization-Assisted amplification (RHAM), a self-developed isothermal amplification molecular detection technology of Pluslife Biotech. The purpose of this study is to evaluate the analytical and clinical performance of this new SARS-CoV-2 POCT method and compare the clinical performance with the EasyNAT Rapid SARS-CoV-2 assay and the Wondfo 2019-nCoV antigen assay. The results of this study are of great significance to clinical practice.

## INTRODUCTION

Acute respiratory infections caused by severe acute respiratory syndrome coronavirus 2 (SARS-CoV-2) still pose a severe public health challenge (1). Sensitive and rapid testing is critical to breaking the transmission chain of SARS-CoV-2. The real-time quantitative polymerase chain reaction (RT-qPCR) method for detecting SARS-CoV-2 RNA in upper respiratory tract samples is widely regarded as the gold standard for clinical diagnosis, given its high sensitivity, specificity, and stability (2). However, RT-qPCR requires sample transportation, nucleic acid extraction and purification, and relies on a thermal cycling instrument with precise temperature control for denaturation, annealing, and other processes (3). Therefore, RT-qPCR is not suitable for point-of-care testing (POCT) due to its time-consuming nature.

In order to achieve rapid on-site screening of SARS-CoV-2, a variety of technologies based on nucleic acid isothermal amplification have emerged, including loop-mediated amplification (LAMP) (4), cross-priming amplification (CPA) (5, 6), rolling circle amplification (7, 8), and recombinase polymerase amplification (RPA) (9). These thermostatic amplification techniques do not involve denaturation, annealing, or other steps and are fast and easy to operate. They are a good choice for clinical POCT implementation. In addition, SARS-CoV-2 testing POCT products based on isothermal amplification technology combined with other technologies have emerged (10–12). The Pluslife Rapid SARS-CoV-2 assay is a new generation of molecular POCT product, utilizing Pluslife’s proprietary RNase Hybridization-Assisted amplification (RHAM) technology (13, 14). The Pluslife Rapid SARS-CoV-2 assay integrates LAMP and RNase HII reporter signal visualization technology and has some advantages, such as low cost and fast results, compared with the traditional POCT products.

The EasyNAT Rapid SARS-CoV-2 assay is a newly developed CPA kit by Ustar Biotechnologies designed to be used with Ustar’s nucleic acid amplification and detection analyzer. The EasyNAT Rapid SARS-CoV-2 assay integrates RNA extraction, RNA purification, and target gene amplification within a single cartridge. It demonstrates high sensitivity and specificity and has been validated for clinical rapid screening of SARS-CoV-2 (15). But the Ustar’s nucleic acid amplification and detection analyzer is neither compact nor portable, limiting its suitability for resource-limited or grassroots settings. The Wondfo 2019-nCoV antigen assay, based on a colloidal gold immunochromatography method, is used to qualitatively detect 2019-nCoV antigen in nasopharyngeal swabs (16). The Wondfo 2019-nCoV antigen assay is simple and fast to operate, making it suitable for home self-tests and large-scale field screening. However, because it detects only surface proteins of the virus, the assay is susceptible to producing false-negative results (17). As a next-generation molecular POCT platform, the Pluslife Mini Dock provides a new molecular detection solution for primary medical institutions and patients with its compact size, rapid processing speed, high accuracy, and cost-effectiveness. Currently, there are no publicly available data comparing the Pluslife Rapid SARS-CoV-2 assay, the EasyNAT Rapid SARS-CoV-2 assay, and the Wondfo 2019-nCoV antigen assay. The objective of this study is to determine the performance characteristics of the Pluslife Rapid SARS-CoV-2 assay and evaluate the consistency of clinical diagnosis with the RT-qPCR. Furthermore, the clinical utility of the Pluslife SARS-CoV-2 assay is evaluated by comparing its performance with the EasyNAT Rapid SARS-CoV-2 assay and the Wondfo 2019-nCoV antigen assay.

## MATERIALS AND METHODS

### The Pluslife Rapid SARS-CoV-2 assay used in this study

The Pluslife Rapid SARS-CoV-2 Card (Pluslife Biotech, Guangzhou, China) was performed on the integrated nucleic acid testing device Pluslife Mini Dock (Pluslife Biotech, Guangzhou, China). The Pluslife Rapid SARS-CoV-2 assay is based on an isothermal amplification method and enzyme-digested probe technology, enabling specific detection of the SARS-CoV-2 N gene and ORF1ab gene. During amplification in the isothermal system, a large number of target gene sequence copies are generated. When the fluorescence-labeled probe hybridizes to the complementary sequences, it is cleaved, resulting in the emission of fluorescent signals. The Pluslife Mini Dock detects and analyzes fluorescence signals, automatically reporting positive, negative, or invalid results.

In this study, the Pluslife Rapid SARS-CoV-2 assay was conducted following the manufacturer’s instructions. First, the disposable sampling swab was inserted into the nucleic acid-releasing agent to release SARS-CoV-2 nucleic acid. Then, 400–450 µL of SARS-CoV-2 nucleic acid release solution was dropped into the SARS-CoV-2 Reaction Card sample tube. After securely screwing the cap of the SARS-CoV-2 Reaction Card sample tube, the air button on top of the card cap was pressed to dissolve the beads in the reaction chip, followed by shaking the card vertically 10 times (within approximately 5 sec). Subsequently, the card was inserted into the integrated nucleic acid testing device, which initiated the testing protocol. The detection process typically took approximately 15–35 min.

### Analytical performance evaluation of the Pluslife Rapid SARS-CoV-2 assay

The limit of detection (LOD) of the Pluslife Rapid SARS-CoV-2 assay was determined using the SARS-CoV-2 nucleic acid quantitative detection reference (BDS Biotechnologies, Guangzhou, China) for absolute quantification. Seven concentrations (800, 600, 500, 400, 300, 200, and 100 copies/mL) were obtained by diluting the SARS-CoV-2 reference (1.0×10^10^ copies/mL). Each concentration was tested in 20 replicates. The LOD was defined as the lowest concentration achieving ≥95% positive detection of SARS-CoV-2 target genes (18).

The cross-reactivity profile of the Pluslife Rapid SARS-CoV-2 assay was systematically evaluated against common respiratory pathogens, including HCoV-229E, HCoV-OC43, Influenza A Virus (IAV), Influenza B Virus (IBV), Epstein-Barr Virus (EBV), Respiratory Syncytial Virus (RSV), Human Metapneumovirus (HMPV), Human Parainfluenza Viruses (HPIVs), and Human Cytomegalovirus (HCMV). Sequence-confirmed nucleic acid-positive clinical specimens for HCoV-229E, HCoV-OC43, IAV, IBV, EBV, RSV, HMPV, HPIVs, and HCMV were obtained from our laboratory archives. Each viral specimen underwent replicate testing, with consistently negative results interpreted as demonstrating the assay’s specificity.

Interference testing for the Pluslife Rapid SARS-CoV-2 assay was assessed in the presence of a low SARS-CoV-2 concentration. Exogenous interfering substances, including oseltamivir (1.875 mg/L), ceftriaxone (1.125 g/L), ribavirin (16.5 mg/L), meropenem (235 mg/mL), tobramycin (30 mg/L), and endogenous interfering substances such as human blood (0.16% vol/vol) were evaluated. All test results were positive, indicating no interference from any of the tested substances.

### Evaluation of the clinical performance of the Pluslife Rapid SARS-CoV-2 assay

#### Clinical samples

This study analyzed retrospectively collected nasopharyngeal swab samples from 197 patients suspected of COVID-19 infection in the outpatient and emergency department of the First Affiliated Hospital of Sun Yat-Sen University between September 2023 and November 2023. These samples were stored at **−**80℃ for subsequent analysis and subjected to SARS-CoV-2 testing using a commercial RT-qPCR kit (DAAN GENE, Guangzhou, China). RT-qPCR served as the gold standard for SARS-CoV-2 detection. Positive and negative samples were first identified based on RT-qPCR results. Each sample was then tested using three rapid SARS-CoV-2 assays: the Pluslife Rapid SARS-CoV-2 assay, the EasyNAT Rapid SARS-CoV-2 assay (Ustar Biotechnologies, Hangzhou, China), and the Wondfo Novel Coronavirus (2019-nCoV) antigen assay (Wondfo Biotech, Guangzhou, China). The clinical performance of these assays was compared.

This study utilized residual biological specimens obtained from clinical settings. Patients with medical orders for COVID-19 testing were invited to participate, and verbal informed consent was obtained from all participants. The study protocol was approved by the Medical Ethics Committee of the First Affiliated Hospital of Sun Yat-sen University.

#### SARS-CoV-2 detection

All assays were performed in accordance with the manufacturer’s instructions. For the RT-qPCR assay serving as the gold standard, commercial kits (DAAN GENE, Guangzhou, China) for SARS-CoV-2 detection were employed. The entire process included nucleic acid extraction, reverse transcription of RNA into complementary DNA, and real-time quantitative PCR amplification using the ABI7500 system (Applied Biosystems, CA, USA). Two hundred microliters of the UTM samples were processed for nucleic acid extraction using the Nucleic Acid Isolation or Purification Kit (DAAN GENE, Guangzhou, China). 5 µL of the nucleic acid elution was used for each RT-PCR reaction. A test result was defined as positive if the cycle threshold (Ct) value for either the ORF1ab or N gene was ≤40.

The EasyNAT Rapid SARS-CoV-2 assay was conducted using Ustar’s nucleic acid amplification and detection analyzer (Ustar Biotechnologies, Hangzhou, China). A 1 mL aliquot of vortex-mixed 2019-nCoV-RNA extraction solution (Ustar Biotechnologies, Hangzhou, China) and 0.5 mL of the sample were added into the sample chamber. The cartridge lid was sealed, and the cartridge was gently shaken to ensure thorough mixing of the solution and sample. Finally, the cartridge was then inserted into the detection analyzer for analysis.

The Wondfo 2019-nCoV antigen assay was performed using the Wondfo antigen test card (Wondfo Biotech, Guangzhou, China). The nasopharyngeal swab sample was inserted into the sample extraction solution and rotated 10 times to ensure optimal dissolution. Then 3–4 drops (80 µL) of the mixture were added to the sample well of the test card. Results were interpreted within a 15–20 min period.

### Statistical analysis

The test results of the Pluslife Rapid SARS-CoV-2 assay, the EasyNAT Rapid SARS-CoV-2 assay, and the Wondfo Novel Coronavirus (2019-nCoV) antigen assay were compared to those of RT-qPCR using positive percentage agreement (PPA), negative percentage agreement (NPA), and overall rate of agreement (ORA). In addition, Cohen’s kappa (*ĸ*) was calculated to assess the overall consistency between each assay and RT-qPCR. Confidence intervals (*CIs*) were computed using the Wilson score method. Statistical analyses were performed using IBM SPSS Statistics 25. *P* < 0.05 was considered statistically significant.

## RESULTS

### Analytical performance of the Pluslife assay

To determine the LOD of Pluslife Rapid SARS-CoV-2 assay, serial dilutions of the standard were prepared at varying concentrations and tested according to the manufacturer’s instructions. The LOD was confirmed as 400 copies/mL (Table 1). To further assess cross-reactivity, the assay was evaluated against other respiratory viruses, including HCoV-229E, HCoV-OC43, IAV, IBV, EBV, RSV, HMPV, HPIVs, and HCMV. The results showed no cross-reactivity with these respiratory viruses, indicating the high specificity for SARS-CoV-2 (Table 2). Interfering substances at concentrations up to those listed in Table 3 showed no interference with the assay performance.

**TABLE 1:** Detection limit of the Pluslife Rapid SARS-CoV-2 assay

Target concentration, copies/mL	No. detected /No. of replicates	Rate (%)
800	20/20	100
600	20/20	100
500	20/20	100
400	20/20	100
300	11/20	55
200	8/20	40
100	0/20	0

**TABLE 2:** Cross-reactivity of the Pluslife Rapid SARS-CoV-2 assay

Target	No. detected /No. of replicates
HCoV-229E	0/2
HCoV-OC43	0/2
IAV	0/2
IBV	0/2
EBV	0/2
RSV	0/2
HMPV	0/2
HPIV	0/2
HCMV	0/2

**TABLE 3:** Interfering substances experiment of the Pluslife Rapid SARS-CoV-2 assay^*a*^

Interfering substance	Concentration	Result
Oseltamivir	1.875 mg/L	+
Ceftriaxone	1.125 g/L	+
Ribavirin	16.5 mg/L	+
Meropenem	235 mg/mL	+
Tobramycin	30 mg/L	+
Human blood	0.16% vol/vol	+

^*a*^ Interfering substance, substance added into the sample with low concentration of SARS-CoV-2; +, positive.

### Clinical performance of three Rapid SARS-CoV-2 assays

A total of 197 nasopharyngeal swab samples were tested for SARS-CoV-2 by RT-qPCR and three rapid SARS-CoV-2 assays: the Pluslife Rapid SARS-CoV-2 assay, the EasyNAT Rapid SARS-CoV-2 assay, and the Wondfo 2019-nCoV antigen assay. Comprehensive concordance metrics relative to the RT-qPCR reference standard are consolidated in Table 4, with detailed Ct-stratified detection rates in Table 5 and discordant analyses in Table 6. The 54 RT-qPCR-confirmed positive samples exhibited Ct values spanning 22–40.

**TABLE 4:** Performance of the three Rapid SARS-CoV-2 assays compared with DAAN RT-qPCR^*a*^

	RT-qPCR		PPA, % (95% CI)	NPA, % (95% CI)	ORA, % (95% CI)	ĸ (95% CI)	P
	Positive	Negative					
Pluslife							
Positive	52	1	96.30	99.30	98.48	0.962	<0.001
Negative	2	142	(87.47–98.98)	(96.14–99.88)	(95.62–99.48)	(0.919–1)	
EasyNAT							
Positive	52	2	96.30	98.60	97.97	0.949	<0.001
Negative	2	141	(87.47–98.98)	(95.04–99.62)	(94.90–99.21)	(0.900–0.998)	
Wondfo							
Positive	44	0	81.48	100.00	94.92	0.865	＜0.001
Negative	10	143	(69.16–89.62)	(97.38–100.00)	(90.90–97.22)	(0.78464–0.94536)	

^*a*^ CI, confidence interval; Ct, cycle threshold.

**TABLE 5:** Detection limit of the three Rapid SARS-CoV-2 assays^*a*^

RT-qPCR Ct	Pluslife		EasyNAT		Wondfo	
	Positive rate, %	95% CI, %	Positive rate, %	95% CI, %	Positive rate, %	95% CI, %
＜25	100.00 (2/2)	34.24–100.00	100.00 (2/2)	34.24–100.00	100 (2/2)	34.24–100.00
25–30	100.00 (16/16)	80.64–100.00	100.00 (16/16)	80.64–100.00	93.75. (15/16)	71.67–98.89
30–35	93.10 (27/29)	78.03–98.09	93.10 (27/29)	78.03–98.09	86.21 (25/29)	69.44–94.50
35–40	100.00 (7/7)	64.57–100.00	100.00 (7/7)	64.57–100.00	28.57 (2/7)	8.22–64.11

^*a*^ CI, confidence interval; Ct, cycle threshold.

**TABLE 6:** Inconsistent results of the four methods^*a*^

Sample	Pluslife	EasyNAT	Wondfo	RT-qPCR	
				N (Ct)	ORF1ab (Ct)
1	+	+	−	28.4697	28.028
2	+	+	−	33.0384	34.1751
3	+	+	−	34.626	34.6737
4	+	+	−	35.0154	36.178
5	−	−	−	35.1521	34.8302
6	−	−	−	35.4529	33.3256
7	+	+	−	35.4807	36.9773
8	+	+	−	37.8547	39.4581
9	+	+	−	38.5115	37.7664
10	+	+	−	38.8048	38.2053
11	+	+	−	Undetermined	Undetermined
12	−	+	−	Undetermined	Undetermined

^*a*^ Ct, cycle threshold; +, positive; −, negative.

Compared with RT-qPCR, the Pluslife Rapid SARS-CoV-2 assay demonstrated a PPA of 96.30% and an NPA of 99.30%. The ORA was 98.48%, and the two assays demonstrated a high level of consistency (*ĸ* = 0.962, *P* < 0.001) (Table 4). The Pluslife assay achieved a 100% positive detection rate for samples with Ct values <30, and a rate >93% for samples with Ct values of 30–40 (Table 5). Three samples exhibited discordant results: two RT-qPCR positive samples tested negative by Pluslife (with Ct values for the N gene or ORF1ab gene exceeding 35), and one RT-qPCR negative sample tested positive by Pluslife (Table 6).

Similarly, compared with RT-qPCR, the PPA and NPA of the EasyNAT Rapid SARS-CoV-2 assay were 96.30% and 98.60%. The ORA was 97.97%, and the results were highly consistent (*k* = 0.949, *P* < 0.001) (Table 4). The EasyNAT assay achieved a 100% detection rate for samples with Ct values <30, 93.10% for samples with Ct values of 30–35, and 100% for samples with Ct values ≥35 (Table 5). Four samples exhibited discordant results: two RT-qPCR-positive samples tested negative by EasyNAT (with Ct values for the N gene or ORF1ab gene >35), and two RT-qPCR-negative samples tested positive by EasyNAT (Table 6).

For the Wondfo 2019-nCoV antigen assay, comparison with RT-qPCR showed a PPA of 81.48% and an NPA of 100.00%. The ORA value was 94.92%, showing good consistency (ĸ = 0.865, *P* < 0.001) (Table 4). The detection rate of the Wondfo assay was 100.00% for Ct values <25; 93.75% for Ct values of 25–30; 86.21% for Ct values of 30–35; and 28.57% for Ct values ≥35 (Table 5). Ten samples showed discordant results between the Wondfo assay and RT-qPCR, with Ct values exceeding 33 for nine of these samples (Table 6).

### Comparison of the three Rapid SARS-CoV-2 assays

Clinical performance comparison of the three rapid SARS-CoV-2 assays on the 54 RT-qPCR-positive samples is detailed in Table 7. Among these positives, the three methods yielded positive results in 52 (96.30%), 52 (96.30%), and 44 (81.48%) cases, respectively. The results showed significant discrepancies (*χ*^2^ = 10.008, *P* < 0.05). The positive rate of the Pluslife assay matched that of the EasyNAT assay. Pairwise comparison revealed statistically significant disparities in detection rates between the molecular assays (Pluslife and EasyNAT) and the antigen-based assay (Wondfo), with both molecular assays exhibiting significantly higher positive rates.

**TABLE 7:** Results of 54 RT-qPCR positive samples detected by the three rapid SARS-CoV-2 assays^*a*^

	Rapid assays (n, %）			*χ* ^2^	P
	Pluslife	EasyNAT	Wondfo		
Positive	52_a_	52_a_	44_b_	10.008	＜0.05
	(96.30%）	(96.30%）	(81.48%)		
Negative	2_a_	2_a_	10_b_		
	(3.70%)	(3.70%)	(18.52%)		

^*a*^ Each subscript letter denoted a subset of Rapid assays categories whose column proportions did not differ significantly from each other at the 0.05 level. The results of Wondfo 2019-nCoV antigen assay were significantly different from those of the EasyNAT Rapid SARS-CoV-2 assay (*P*＜0.05).

## DISCUSSION

Coronavirus disease 2019 (COVID-19) remains one of the leading causes of epidemic respiratory infections. Currently, the most widely accepted diagnostic test for SARS-CoV-2 is still based on the gold standard method RT-qPCR (19). However, laboratory RT-qPCR testing remains inaccessible in lots of countries and cannot be widely implemented in resource-limited settings (20). Although rapid antigen tests offer convenience, their sensitivity is inferior to molecular assays (21, 22). In recent years, emerging isothermal amplification technologies (eg., CPA, RPA) have enabled portable molecular diagnostics, yet most commercial systems remain cost-prohibitive for point-of-care use. This study evaluated the performance of the novel Pluslife Rapid SARS-CoV-2 assay, a palm-sized POCT platform, using RT-qPCR as the reference method, with comparative analysis against established assays: the EasyNAT Rapid SARS-CoV-2 molecular POCT and the Wondfo 2019-nCoV GICA.

The Pluslife Rapid SARS-CoV-2 assay exhibited a low LOD of 400 copies/mL. It showed no cross-reactivity with other common respiratory viruses and no interference from tested substances, demonstrating a good diagnostic performance. For clinical samples, there was a good consistency (*ĸ* = 0.962, *P* <0.001) between the Pluslife Rapid SARS-CoV-2 assay and RT-qPCR. Using the Pluslife Rapid SARS-CoV-2 assay, the positive samples can be determined as fast as 7 min, while negative samples can be confirmed in about 35 min (Table 8). Its rapid, accurate, user-friendly, and cost-effective features align with POCT criteria (23, 24), making it suitable for primary care and resource-limited settings.

**TABLE 8:** Characteristics of the four SARS-CoV-2 detection devices^*a*^

	Pluslife SARS-CoV-2 assay	EasyNAT SARS-CoV-2 assay	Wondfo 2019-nCoV antigen assay	DAAN SARS-CoV-2 assay
Device	Pluslife Mini Dock	Ustar UC0108	/	ABI7500
Manufacturer	Pluslife Biotech, Guangzhou, China	Ustar Biotech, Hangzhou, China	Wondfo Biotech, Guangzhou, China	DAAN GENE, Guangzhou, China
Technology	RHAM	CPA	GICA	RT-qPCR
Target	N and ORF1ab	N and ORF1ab	antigen	N and ORF1ab
LOD	400 copies/mL	200 copies/mL	/	200 copies/mL
Hands-on time	1–2 min	＜5 min	1–2 min	＞1 h
Time-to-result	7–35 min	–40 min	15–20 min	＞1 h
Dimension	101×86×63 mm	460×470×390 mm	/	340×450×490 mm
Weight	240 g	33,000 g	/	34,000 g
Cost per device	$2,000–5,000	$35,000–45,000	/	$60,000–70,000
Cost per test	$3–8	$4–10	$1–3	$5–15

^*a*^ RHAM, RNase Hybridization-Assisted amplification; CPA, Cross-priming amplification; GICA, Gold Immunochromatography; RT-qPCR, real-time quantitative polymerase chain reaction; /, not applicable.

In addition, this study represents the first comparative analysis of the Pluslife Rapid SARS-CoV-2 assay, the EasyNAT Rapid SARS-CoV-2 assay, and the Wondfo 2019-nCoV antigen assay. Using RT-qPCR as the gold standard, the PPA of the Pluslife assay achieved 96.30%, which was in line with 96.30% of the EasyNAT assay and superior to 81.48% of the Wondfo assay. For NPA, the Pluslife assay demonstrated an accuracy of 99.30%, surpassing the EasyNAT assay (98.60%). It is worth mentioning that among the 197 clinical samples, the ORA of the Pluslife assay was 98.48%, exceeding both the EasyNAT assay (97.97%) and the Wondfo assay (94.92%), indicating superior clinical reliability. For RT-qPCR positive samples, statistical analysis revealed significant differences among the three assays (*χ*^2^ = 10.008, *P* < 0.05). Pairwise comparisons revealed significant discrepancies between both molecular assays (Pluslife, EasyNAT) and the Wondfo antigen assay.

Notably, while the Pluslife and EasyNAT assays demonstrated equivalent efficacy in detecting positive samples, the EasyNAT assay requires an expensive benchtop instrument and sophisticated laboratory infrastructure. In contrast, the Pluslife Mini Dock is extensively smaller and more economical, with faster results (positives: ~7 min vs 40 min). Moreover, the Pluslife assay demonstrates clear economic superiority in per-device and per-test costs (Table 8), making it more portable, faster, and cost-effective. Besides, the difference between the Pluslife assay and the Wondfo assay was statistically significant. The PPA of the Pluslife assay was about 15% higher than that of the Wondfo assay. When Ct values for the N gene or ORF1ab gene exceeded 35, the Wondfo assay was more likely to miss the positive samples.

The emergence of molecular POCT plays a critical role in the rapid diagnosis and differentiation of respiratory diseases (25–27). Between the two rapid SARS-CoV-2 molecular POCT assays compared in this study, the Pluslife assay showed better performance than the EasyNAT assay, featuring good detection performance, compact size, low cost, simple operation, and rapid result delivery. Moreover, unlike the Wondfo assay, which detects viral surface proteins (28, 29), the Pluslife Mini Dock amplifies SARS-CoV-2 RNA exponentially, significantly reducing the likelihood of false-negative results within minutes.

There are several limitations of this study. First, RT-qPCR results—rather than clinical disease diagnosis—were used as the gold standard. RT-qPCR reliability may be influenced by incomplete clinical follow-up data. RT-qPCR results might not accurately reflect the SARS-CoV-2 carrying status of samples (30). PPA and NPA are inherently better suited for consistency assessment between two diagnostic methods. Therefore, instead of sensitivity and specificity, the PPA and NPA were used to characterize the clinical performance of the rapid SARS-CoV-2 assay compared with RT-qPCR (25). Additionally, viral genome sequencing was not performed, precluding assessment of variant impacts. Moreover, the sample size was small, and most of the Ct values for positive samples exceeded 35. This may explain why the PPA value was not as high as the 99% PPA value provided by Laura Herrmann (31). Finally, clinical samples were not tested by all four methods concurrently, and SARS-CoV-2 in samples stored at **−**80℃ may have degraded during repeated freeze-thaw processes.

In summary, the Pluslife assay shows good consistency with RT-qPCR. Compared with the EasyNAT assay, the Pluslife assay exhibits the same excellent detection efficiency of SARS-CoV-2 positive samples, with smaller size, lower cost, and faster detection speed. Compared with the Wondfo assay, the clinical performance of the Pluslife assay is more prominent, with a lower false-negative rate. The Pluslife Mini Dock’s compact design, portability, simple operation, rapid detection, capacity, and cost-effectiveness support its use for SARS-CoV-2 screening in resource-limited settings.
